# Case report: Radiopaque mandibular lesions in three dogs

**DOI:** 10.3389/fvets.2024.1529669

**Published:** 2025-01-15

**Authors:** Senni Vesterinen, Hanna-Kaisa Sihvo, Niina Airas, Helena Kuntsi

**Affiliations:** ^1^Anident Veterinary Clinic, Kirkkonummi, Finland; ^2^Solumo Pathologists Ltd, Helsinki, Finland

**Keywords:** radiopaque, mandible, canine, idiopathic osteosclerosis, cone beam computed tomography

## Abstract

Radiopaque lesions of the mandible are occasional findings in dental radiographs in dogs. The different diagnoses of densely sclerotic lesions in humans include odontoma, idiopathic osteosclerosis, condensing osteitis, hypercementosis, osteoma, osteoblastoma, and oral exostosis. Publications on many of these conditions in dogs are scarce. This clinical report describes three young adult dogs with radiologically and histologically similar radiopaque mandibular lesions that had either displaced or narrowed the mandibular canal. One dog showed symptoms that could have been consistent with neurological pain due to the lesion. Diagnostics included clinical examination, dental radiographs, cone beam computed tomography and histology. Radiographically and clinically, the lesions resembled human idiopathic osteosclerosis. Histology alone did not reveal a definitive diagnosis, but combining histology with clinical and radiographic data, the most likely diagnosis was idiopathic osteosclerosis. The dogs returned for follow-ups 6 months after the surgeries, and there was no evidence of additional growth in the remaining lesions.

## Introduction

Radiopaque lesions of the mandible are occasional findings in dental radiographs in dogs (1) but, to the authors' knowledge, histological diagnosis remains unpublished. Radiographic description of the lesions includes location, shape and size, attenuation pattern, margin characteristics, relation to the adjacent teeth, and possible evidence of surrounding cortical expansion or radiolucent halo (2, 3). In dental radiographs, the description of the location of the lesions is based on a two-dimensional view, but advanced, three-dimensional imaging, such as cone-beam computed tomography (CBCT), allows identifying the exact location of the lesions and their position in relation to the mandibular canal. Obtaining biopsies for histopathological examination may be challenging due to the location near the inferior alveolar neuro-vascular bundle.

The human medical texts categorize the differential diagnosis of radiopaque mandibular lesions based on their attenuation patterns into densely sclerotic, ground-glass, and mixed lytic-sclerotic patterns (2). Additionally, they classify lesions by etiology into odontogenic and non-odontogenic origins (2). Table 1 presents the differential diagnoses according to these categories (1–4). The densely sclerotic lesions are all benign and radiographically relatively homogenous, resembling the radiopacity of cortical bone or tooth structures (2). This article focuses on densely sclerotic lesions only.

**Table 1:** Differential diagnoses of radiopaque jaw lesions.

	**Densely sclerotic lesions**	**Groung-glass lesions**	**Mixed lytic-sclerotic lesions**
Odontogenic	Odontoma (D)	Cemento-ossifying fibroma (D)	Developing odontoma (D)
	Cemento-osseus dysplasia		Calcifying epithelial odontogenic tumor (D: amyloid-producing ameloblastoma/amyloid-producing odontogenic tumor)
	Cementoblastoma (D)		
	Condensing osteitis (D)		
	Idiopathic osteosclerosis (D)		
	Hypercementosis (D)		
Non-odontogenic	Osteoma (D)	Fibrous dysplasia (D)	Osteosarcoma (D)
	Osteoblastoma	Paget disease of bone	Chronic osteomyelitis (D)
	Oral exostosis	Renal osteodystrophy (D)	Osteonecrosis (D)
			Metastases (D)

The entities reported in dogs are marked with (D).

Densely sclerotic odontogenic lesions include odontoma, cemento-osseous dysplasia, cementoblastoma, condensing osteitis, idiopathic osteosclerosis and hypercementosis (2, 3). Odontomas are hamartomas that constitute from dentinal tissues, and they exist in two subtypes, compound and complex (3). Radiographically, compound odontomas resemble multiple radiopaque tooth-like structures known as denticles (4). Complex odontomas present as a singular, heterogenous, densely sclerotic lesion with a thin radiolucent halo (3). Veterinary literature features reports of both odontoma types with singular case reports and series with three cases (4–7).

Cemento-osseus dysplasia is a form of benign, intraosseous, proliferative fibro-osseous lesions in which a fibrous connective tissue matrix, containing abnormal bone or cementum, replaces normal bone (3). Cemento-osseus dysplasia is more common in the mandible and can manifest either as a periapical or focal single lesion or florid multiple lesions (8). These lesions are typically asymptomatic but can also be symptomatic and need treatment (3). Radiographically, these lesions are densely sclerotic, homogenous, well-defined, variable in size, may expand the cortical bone, and typically have a radiolucent halo (3). Veterinary literature reports other types of proliferative fibro-osseus lesions (ossifying fibroma and fibrous dysplasia) in dogs (9), but to the authors knowledge, no publications of cemento-osseous dysplasia exist.

Cementoblastomas are rare benign tumors, arising from cementoblasts (4, 8). Radiographically, they appear as densely sclerotic or mixed radiolucent and radiopaque lesions attached to tooth roots (3). Cementoblastomas may expand cortical bone or destruct adjacent tooth roots (3) and cause low-grade pain (10). Literature includes two presumptive cases of cementoblastomas in dogs (4).

Condensing osteitis, i.e., sclerosing osteitis, is a form of periapical inflammation due to periodontal or endodontal disease (11, 12). Radiographically, it presents as a densely sclerotic, ill-defined periapical lesion of a necrotic tooth, with possible widening of the periodontal ligament space of the attached tooth root (3). Veterinary literature provides information about this condition in dogs but to the authors knowledge, no studies in dogs (4, 12).

Idiopathic osteosclerosis is also called enostosis, osteosclerosis focus, periapical osteopetrosis, dense bone island, or bone scar (3, 13). The pathogenesis of these lesions remains unknown, but they may represent developmental variants (2). Idiopathic osteosclerosis is most commonly asymptomatic, radiographically well-defined, densely sclerotic lesion with variable size, and without a radiolucent rim or cortical expansion (2, 3, 13). The periphery of the lesion may blend with the trabeculae of the surrounding bone (3). It may or may not be adjacent to vital tooth roots and requires only monitoring (2, 13). These lesions can, however, cause tooth resorption, disrupt tooth eruption, complicate orthodontic treatment, or cause neuralgic pain if occluding the mandibular canal (13). The prevalence of idiopathic osteosclerosis in humans varies from 1.96% to 26.9%, and it is more common in the mandible than in the maxilla (13). In dogs textbooks describe mandibular lesions radiographically resembling human idiopathic osteosclerosis (1, 11, 14) and a single case report exists, in which the diagnosis relies solely on the appearance in dental radiographs without the use of three-dimensional imaging or histopathology (1).

Hypercementosis is also called cemental dysplasia (10). This condition may affect a single tooth or multiple teeth and involves the production of excessive amount of cementum that exceeds the physiological limits (15), but its etiology remains poorly understood (15). The human medical literature lists several possible causes for hypercementosis, including intensive masticatory effort, carious lesions, apical periodontitis, tooth impaction, periodontal disease, concrescence, super eruption, drugs, and systemic disease such as Paget's disease or a combination of these (15). Radiographically, hypercementosis is a smooth or occasionally irregular, tooth-associated density either affecting the entire root or being local, most commonly affecting the apical third of the root. The periodontal ligament space remains detectable (11, 15). Veterinary textbooks describe cases of hypercementosis in dogs (11, 14), but this condition remains poorly defined in both humans and dogs.

Veterinary texts describe an additional type of hypercementosis in horses and other herbivores, but not in humans or dogs. In nodular hypercementosis, highly mineralized cementum is either attaching the tooth root or as a sole density apart from the roots (4, 16). Another name for nodular hypercementosis in the literature is cementoma, which can be misleading since it is not a neoplastic lesion (4, 16).

Densely sclerotic, non-odontogenic lesions include osteoma, osteoblastoma, and oral exostosis (2, 3).

Osteomas are benign, slowly progressing, painless bone tumors or sometimes categorized as hamartomas (3, 4). Radiographically, they are homogenously sclerotic, well-defined lesions without a radiolucent halo and either expand the bone cortex or are pedunculated (17). They are most common in the caudal mandible and may displace adjacent structures such as teeth (3). The veterinary literature features several reports and a case series with sex cases of osteomas in dogs (4, 17, 18). Osteblastomas are true benign bone tumors arising from osteoblasts (3). Radiographically and histologically, they resemble cementoblastomas but are not attached to a tooth root (3, 4). Veterinary reports of osteoblastomas are scarce (4).

Exostoses are hamartomas of mostly cortical bone, arising from the bone surface and typically due to inflammation or trauma, and self-limiting by growth (11, 17). Radiographically, they may be difficult to differentiate from osteomas (4). To the authors' knowledge, veterinary literature does not report oral exostosis in a dog.

To better understand the location and structure of the radiopaque mandibular lesions in dogs, we present here three cases with clinical and radiographic data, including cone beam computed tomography, and histopathology of the lesions.

## Case reports

### Case 1

A 3-year-old male American Akita presented due to a radiopaque lesion in the left mandible. The lesion had been an incidental finding in full-mouth dental radiographs during a routine periodontal treatment, and there were no oral or dental symptoms. The dog had hypothyroidism, treated with levothyroxine. During the general physical examination, the only significant finding was alopecia on the ventral side of the neck. The extraoral and oral exam, along with dental charting under anesthesia, revealed a supernumerary right mandibular third molar tooth but no other significant findings.

**Figure 1:**
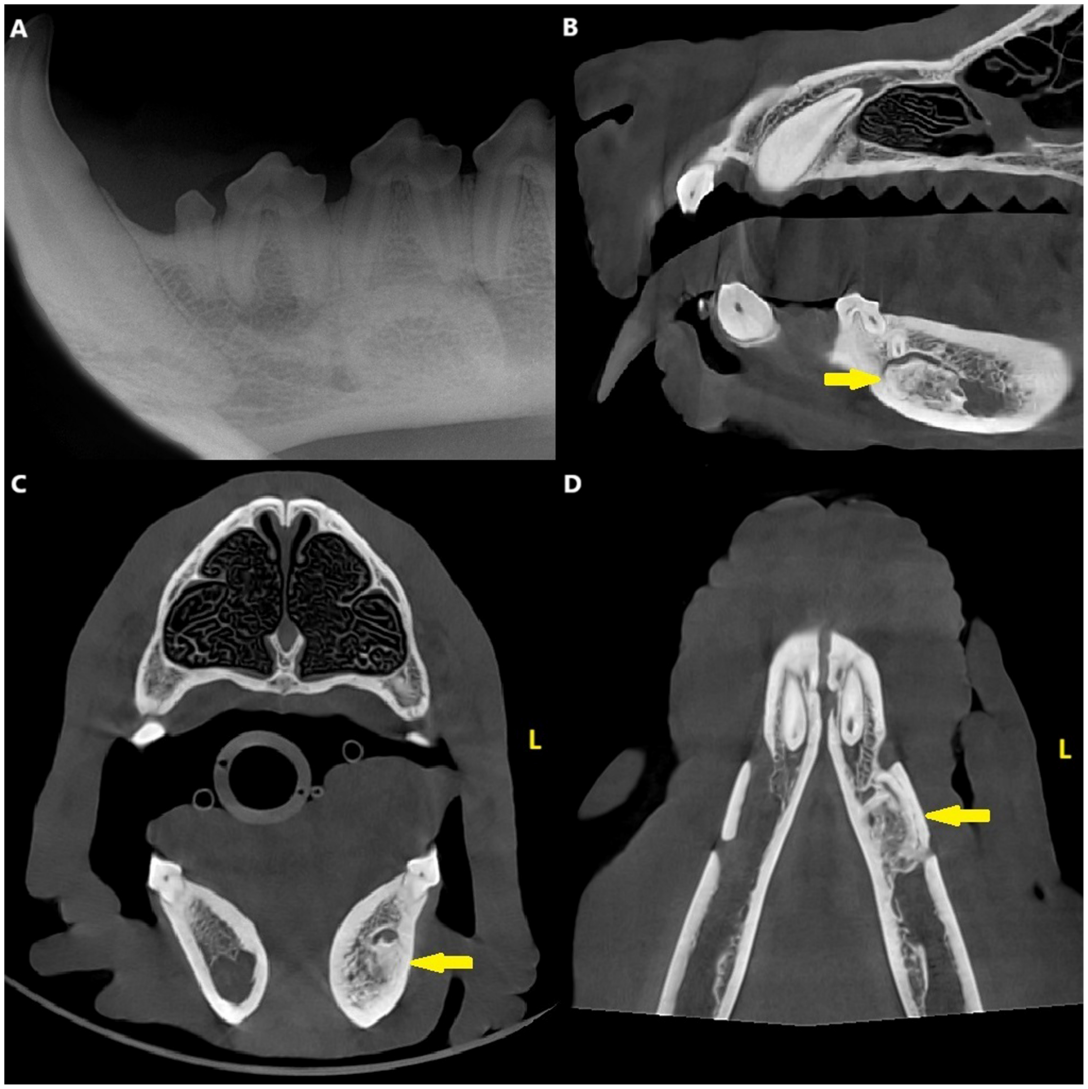
Case 1: Mandibular dental radiograph **(A)** and sagittal **(B)**, transverse **(C)** and dorsal **(D)** planes of the cone beam computed tomography showing the variably sclerotic, oval-shaped lesion (arrow) positioned apically to the left mandibular third premolar tooth.

Dental radiographs and CBCT revealed a variably sclerotic, oval-shaped lesion with a diameter of 17 × 8 × 9 mm apically to the left mandibular third premolar tooth, overlapping with the mandibular canal and displacing the inferior alveolar neuro-vascular bundle both dorsally and lingually. The edges of the lesion were partly smooth and well-defined and partially irregular and ill-defined, with the periphery of the lesion blending with the trabeculae of the mandibular bone. There was no radiolucent halo around the lesion nor cortical expansion. The lesion did not appear to be in direct contact with the teeth, and the periodontal ligament space surrounding the roots of the left second, third, and fourth premolar teeth was uniformly wide (Figure 1).

Surgery consisted of a biopsy of the lesion by creating a bone window to the buccal cortex of the mandible, using a dental burr and a piezo surgery unit (Piezotome Cube, Acteon). Hemorrhage from the inferior alveolar vessels was controlled with ligation. Histologically, the samples consisted of poorly cellular eosinophilic matrix with lacunae, which were empty or contained a faintly visible nucleus. Occasional thin basophilic lines were detected among the matrix (resting and reversing lines). Signs of inflammation or infectious agents were not present.

At the re-check, 6 months post-surgery, the dog was asymptomatic, and CBCT and dental radiographs revealed no changes of the lesion compared to postoperative radiographs.

### Case 2

A 1-year-old female Labrador retriever presented due to fractures in both maxillary fourth premolar teeth. At presentation, the dog was generally healthy and asymptomatic. The general physical examination revealed no abnormal findings aside from the fractured teeth. The extraoral and oral exam, along with dental charting under anesthesia, revealed uncomplicated crown fractures in both maxillary fourth premolars but no other significant findings.

**Figure 2:**
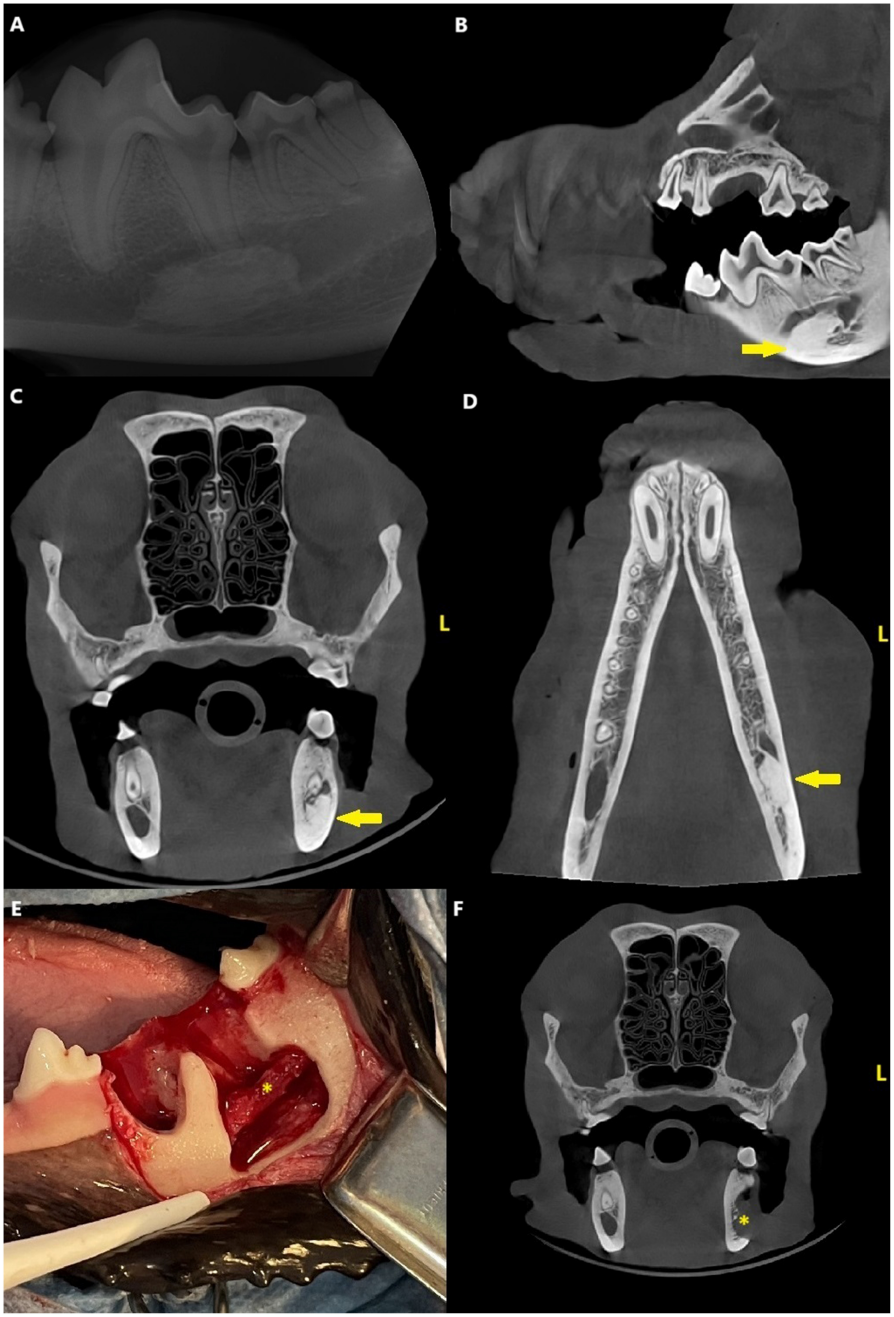
Case 2: Mandibular dental radiograph **(A)** and sagittal **(B)**, transverse **(C)** and dorsal **(D)** planes of the cone beam computed tomography showing the densely sclerotic, homogenous, oval-shaped lesion (arrow) apical to the distal root of the left mandibular first molar tooth. Clinical photograph **(E)** from the surgery of the same patient and a transverse plane **(F)** of the cone beam computed tomography from the postoperative images showing the more anatomical placement of the inferior alveolar bundle (asterisk).

Dental radiographs and CBCT revealed a densely sclerotic, homogenous, oval-shaped lesion with a diameter of 17 × 8 × 8 mm apical to the distal root of the left mandibular first molar tooth, overlapping with the mandibular canal and displacing the inferior alveolar neuro-vascular bundle both dorsally and buccally. The edges of the lesion were smooth and well-defined, and there was no radiolucent halo around the lesion nor cortical expansion. The lesion was apical to the distal root of the left first molar tooth, but the periodontal ligament space surrounding the root was uniformly wide (Figure 2).

The owner was hesitant to proceed with further diagnostics at this time, so the plan was to monitor the lesion with CBCT after 6 months. The uncomplicated crown fractures were restored.

After 3 months, the dog presented with right-sided infraorbital swelling and clinical signs of pain due to periapical pathology of the right maxillary fourth premolar tooth. The tooth was surgically extracted and dental radiographs and CBCT were obtained to monitor the mandibular lesion. The lesion had slightly increased in size and was now 18 × 8 × 8 mm.

At a 7-month recheck, the dog presented with clinical signs of scratching the left side of the jaw. Radiographs and CBCT revealed no changes in the size and shape of lesion compared to 4 months previously. Due to the suspected neurological pain, the plan was to proceed with further diagnostics of the lesion.

**Figure 3:**
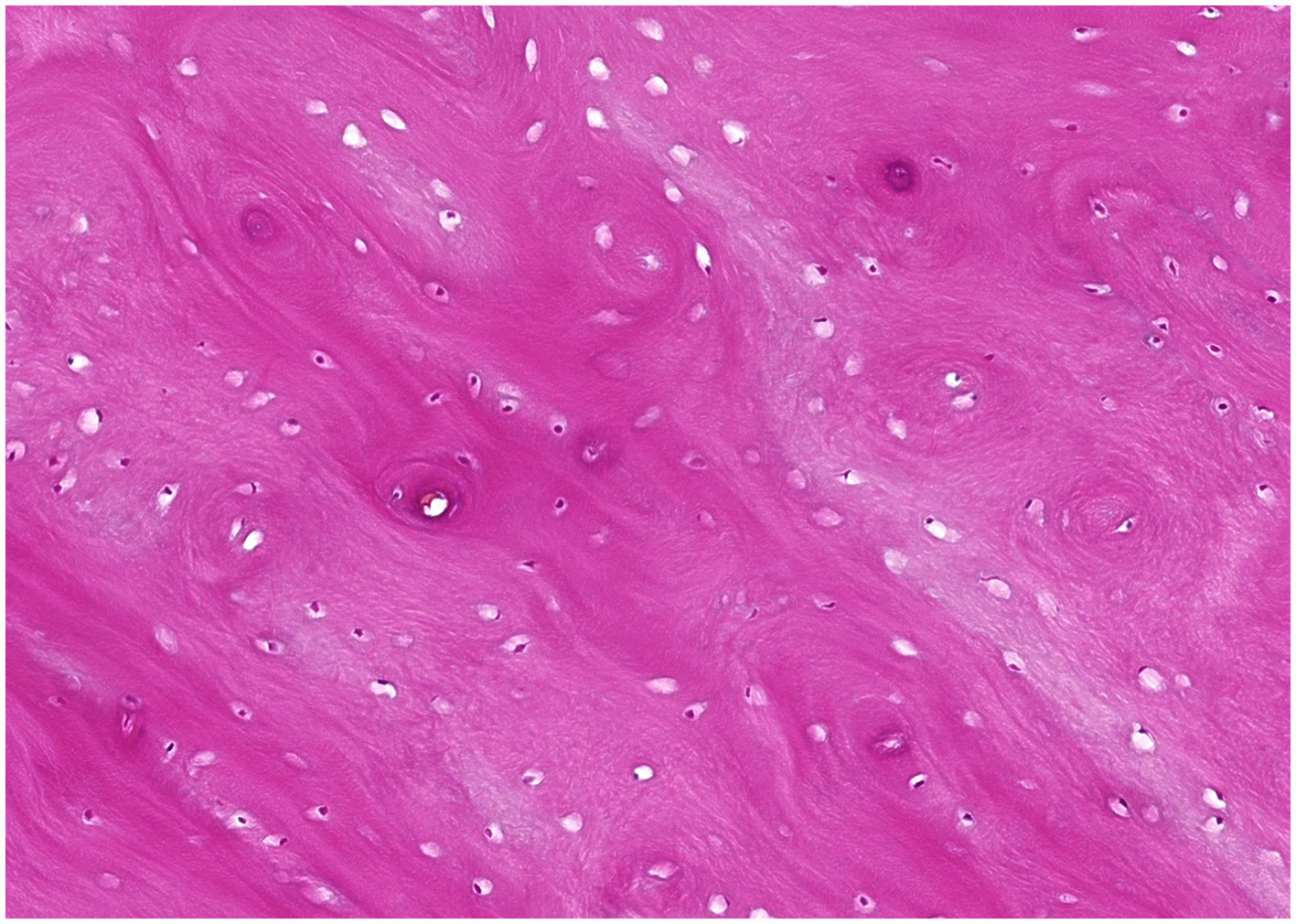
Microscopic image of a hematoxylin and eosin-stained tissue section of Case 2. The sample is consistent with osseous or cemento-osseous tissue and comprised of extracellular matrix containing lacunae variably populated by osteocytes. The extracellular matrix lamellae are arranged in parallel streams or occasional concentric whorls resembling osteonal architecture.

Surgery consisted of extraction of the left first molar tooth, creation of a buccal bone window, and partial ectomy for biopsy of the mandibular lesion using a dental burr and a piezo surgery unit (Piezotome Cube, Acteon). The inferior alveolar neurovascular bundle remained intact and appeared to be in a more normal location after the surgery (Figure 2). Histopathological examination showed poorly cellular, dense eosinophilic matrix with multifocal lacunae and resting and reversal lines. Multifocally, the matrix formed osteon-like circular structures around an empty canal. Some canals included erythrocytes. Signs of inflammation or infectious agents were not present (Figure 3).

At the re-check, 6 months post-surgery, the dog was asymptomatic, and CBCT revealed no changes of the lesion compared to postoperative images.

### Case 3

A 2-year-old male German Shepherd presented due to a radiopaque lesion in the left mandible. The lesion had been an incidental finding in full-mouth dental radiographs during routine periodontal treatment, and there were no oral or dental symptoms. At presentation, the dog was generally healthy and asymptomatic. There were no abnormal findings in the general physical examination. The extraoral and oral exam, along with dental charting under anesthesia, revealed no significant findings.

**Figure 4:**
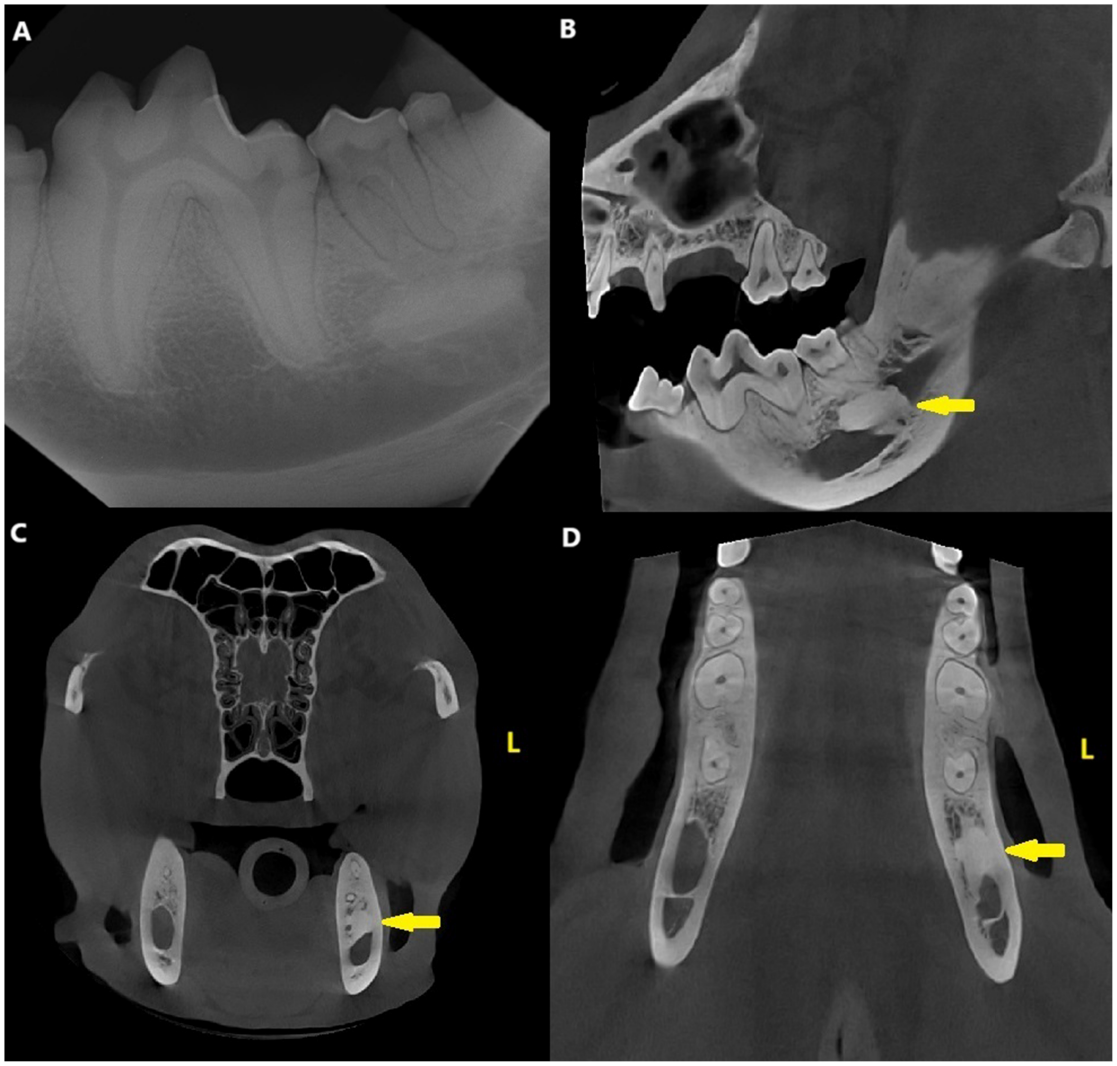
Case 3: Mandibular dental radiograph **(A)** and sagittal **(B)**, transverse **(C)** and dorsal **(D)** planes of the cone beam computed tomography showing the variably sclerotic, oval-shaped lesion (arrow) apically to the left mandibular second molar tooth.

Dental radiographs and CBCT revealed a variably sclerotic, oval-shaped lesion with a diameter of 15 × 14 × 7 mm apically to the left mandibular second molar tooth, overlapping with the mandibular canal. The diameter of the mandibular canal at the lesion site was narrower than the surrounding canal, measuring 4 mm, compared to the 8 mm of the surrounding canal. The edges of the lesion were partly smooth and well-defined and partially irregular and ill-defined, with the periphery of the lesion blending with the trabeculae of the mandibular bone. There was no evidence of a radiolucent halo around the lesion nor cortical expansion. The lesion did not appear to be in direct contact with the teeth, and the periodontal ligament space surrounding the roots of the left second molar tooth was uniformly wide (Figure 4).

Surgery consisted of extraction of the left second molar tooth, creation of a buccal bone window, and a biopsy with partial ectomy of the mandibular lesion using a dental burr and a piezo surgery unit (Piezotome Cube, Acteon). The inferior alveolar neurovascular bundle remained intact and appeared to be in a more normal location after the surgery. Histopathological changes of this case were similar to what was described above for case 2.

At the re-check, 6 months post-surgery, the dog was asymptomatic, and CBCT revealed no changes of the lesion compared to postoperative images.

## Discussion

These three cases shared many similarities: all dogs were young adults, and the lesions were similar both radiographically and histologically. Among the differential diagnoses, we could rule out many possibilities according to clinical, radiographic and histologic findings. Compound odontomas present with a pathognomonic radiographic appearance with denticles. Complex odontomas have a radiolucent halo (3) and histologically, present with ectomesenchymal tissue (4). Both of these were lacking in our cases. Contrary to our cases, cemento-osseus dysplasia has a characteristic radiolucent halo, tooth association, and loss of lamina dura of the affected tooth (3). Cementoblastomas are intimately tooth-associated (4), have a radiolucent halo, and typically cause resorption in the adjacent tooth root (3)—none of these findings were present in our cases. Hypercementosis is also typically attached to the tooth root (15). Osteoma typically affects older humans and dogs (3, 17), with the lesions in the human mandible being exophytic and radiographically uniformly radiopaque (3). Exostoses are also exophytic and arise from the bony surface (3). In our cases, the radiopacities were within the bone. Osteoblastomas typically have a radiolucent halo, cause bone expansion and resorption in the adjacent tooth roots (2). None of these characteristics were met in any of our cases. Condensing osteitis is always due to periapical inflammation (11), but in our three cases, there were no signs of endodontically or periodontally compromised teeth and signs of inflammation were lacking in the histopathological examinations.

In humans, idiopathic osteosclerosis lesions are most common in young adults and usually in the mandible, often overlapping with the mandibular canal (13). They are usually asymptomatic, but in some cases, can cause neurological pain (13). The main diagnostic tool in humans is CBCT (13). The human patient meets the criteria if patient is under 40 years old, the lamina dura and periodontal ligament space are intact and there is absence of endodontic issues, radiolucent halo surrounding the lesion, radiolucency within the lesion, bone expansion and history of tooth extractions in the area (13). All our three cases fit each of these criteria: all dogs were young adults, all lesions were in the mandible, and all radiographic criteria were met.

Histology alone, however, did not provide definitive diagnosis in our cases. Odontoma as the only definitive rule out. Biopsy findings in all three cases were consistent with cemento-osseus tissue devoid of interosseus spaces, suggestive of sclerosis. Other tooth structures, such as dentin, which would have been pathognomonic for odontoma, were absent. The other densely sclerotic odontogenic entities are challenging to differentiate histologically, especially when adjacent structures such as teeth are not included in the biopsy and thus the exact location of the lesion could not be confirmed histologically.

Combining information from clinical picture, imaging, and histopathological examination, the diagnosis of all our three cases is idiopathic osteosclerosis.

Idiopathic osteosclerosis in humans typically does not require treatment, but it is advisable to schedule follow-ups (13). Even though they commonly appear to remain static in size (3, 13), they may occasionally grow or cause tooth resorption, disrupt tooth eruption, complicate orthodontic treatment, cause neuralgic pain, or partial paresthesia (13, 19). The most likely cause of neuralgic pain is presence of the lesion in the mandibular canal, causing discomfort to the inferior alveolar nerve (13). Without three-dimensional imaging, evaluation of the displacement or narrowing of the mandibular canal may be impossible. In each of our three cases, we opted for surgical biopsies due to the lesion causing displacement or severe narrowing of the mandibular canal. In case 2, the dog also presented with scratching of the mandible which could have been a sign of neuralgic pain or discomfort even though in dogs it is impossible reliably diagnose intermittent pain or paresthesia. Surgery of the lesions consisted of partial ectomy as biopsy. It proved challenging to visually distinguish the lesion from the surrounding normal bone. The goal was to preserve the inferior alveolar neurovascular bundle and enhance its anatomical position. In humans, given the risk of recurrence of these lesions, the surgical treatment, if indicated, usually aims at complete excision with safety margins (13, 20). One human article reports recurrence of the lesion 5 months after the surgical resection (20). In our cases, we only aimed at retrieving enough tissue for histopathology and freeing the neurovascular bundle, and after 6 months, no regrowth was evident in any of the three cases.

Even though radiological and histological findings of all these three cases were consistent with idiopathic osteosclerosis, considering the information from the human medicine, it is likely that multiple different diagnoses of mandibular radiopacities are also possible in dogs. Limitations of our study are the small number of the cases and retrospective nature of the study. Prospective studies with more cases utilizing not only clinical signs and dental radiographs, but also three-dimensional imaging and histological diagnosis are necessary to better understand the pathology of these lesions.

A practical recommendation for veterinary general practitioners encountering these lesions during routine imaging is to acquire careful clinical examination and three-dimensional imaging with regular follow-ups to monitor clinical signs and the behavior of the lesions. In cases with suspect neuralgic pain, growth of the lesion, or displacement or narrowing of the mandibular canal, a biopsy obtained by a specialist is indicated. The biopsy may require surgical teeth extractions and creating a bony window at a critical proximity to the inferior alveolar neurovascular bundle so utilization of advanced techniques such as piezoelectric surgery are necessary. A lack of a reliable way to diagnose neuralgic pain and paresthesia in dogs complicates the clinical decision making in weighing the benefits and possible risks of proceeding with diagnostics.

## Data Availability

The original contributions presented in the study are included in the article/supplementary material, further inquiries can be directed to the corresponding author.
